# Relationships between Muscle Architecture of Rectus Femoris and Functional Parameters of Knee Motion in Adults with Down Syndrome

**DOI:** 10.1155/2016/7546179

**Published:** 2016-11-08

**Authors:** Maria Stella Valle, Antonino Casabona, Marco Micale, Matteo Cioni

**Affiliations:** ^1^Gait and Posture Motion Analysis Laboratory, Department of Biomedical and Biotechnological Sciences, University of Catania, 95125 Catania, Italy; ^2^Physical Medicine and Rehabilitation Residency Program, Department of Biomedical and Biotechnological Sciences, University of Catania, 95125 Catania, Italy

## Abstract

This study was designed to measure* in vivo* muscle architecture of the rectus femoris in adults with Down syndrome, testing possible relationships with functional parameters of the knee motion. Ten adults with Down syndrome and ten typically developed participated in the study. Pennation angle and thickness of the rectus femoris and subcutaneous layer of the thigh were measured via ultrasound imaging. Knee kinematics and electromyographic activity of the rectus femoris were recorded during free leg dropping. Muscle thickness was reduced and subcutaneous layer was thicker in persons with Down syndrome with respect to typically developed adults, but there were no differences in the pennation angle. The area of the rectus femoris EMG activity during the leg flexion was greater in Down syndrome with respect to typically developed adults. The leg movement velocity was lower in Down people than in controls, but the knee excursion was similar between the groups. Functional parameters correlated with pennation angle in the persons with Down syndrome and with muscle thickness in typically developed persons. The description of muscle architecture and the relationships between morphological and functional parameters may provide insights on the limits and the opportunities to overcome the inherent biomechanical instability in Down syndrome.

## 1. Introduction

Down syndrome (DS) is a genetic disease that is typically manifested by mental retardation, delayed motor development, perceptual-motor deficits, and hypotonia with ligamentous laxity [1]. Low muscle strength or weakness was reported and quantified for arm and leg muscles in adults, children, and adolescents with DS [2, 3]. Cioni and colleagues [3] demonstrated that knee extensor muscle weakness becomes increasingly evident during adolescence due to the failure to increase muscle strength that occurs physiologically after puberty. Furthermore, a positive correlation was found between leg muscle strength and bone mineral density, indicating that an active lifestyle that improves muscular strength should be instituted in persons with DS to avoid the development of osteoporosis [4]. On the basis of these initial studies, training protocols using a treadmill walking program were undertaken to improve muscle strength in adolescents with DS [5], resulting in positive effects on the development of muscle strength in the lower limbs and physical activity levels.

The underlying causes of muscle weakness in DS are not yet fully known. Neural abnormalities of the motor cortex were reported in persons with DS [6, 7]. Premature ageing of the neuromuscular junction [8], overinhibition of calcineurin, a key mediator in the hypertrophic response of muscles during development [9], and ultrastructural abnormalities of the mitochondria and myonuclei of quadriceps muscle fibers, similar to those observed in age-related sarcopenic muscles [10], were observed in animal models of DS.

There is little information on the causes of muscle hypotonia in DS, which is particularly evident at birth and throughout life. In a recent study in persons with DS and muscle hypotonia, Dey and colleagues [11] reported on mutations in the specific collagen molecule (COL6A3), an essential component for maintaining muscle integrity. In the aforementioned study, a simple nucleotide polymorphism rs 2270669 “C” in COL6A3 may be considered a risk factor for muscle hypotonia in DS.

Several studies reported that persons with DS overcome their mechanical impairments accomplishing novel muscular strategies [12–14]. For example, from adolescence to adult age, persons with DS develop adaptive neuromuscular responses to muscle hypotonia and ligament laxity following the sudden release of leg [14]. In fact, adults with DS showed an increase in reflex muscle activity with respect to adults without DS during the leg dropping [13].

In spite of the evident clinical interest, no published studies report on* in vivo* anatomical architecture of the striate muscle in DS. We hypothesized that an abnormal architecture of muscle or surrounding tissues may be present in DS. Morphological characteristics of rectus femoris (RF) muscle and surrounding tissues were investigated in a group of young adults with DS using ultrasound (US) imaging to identify new anatomical insights* in vivo*. Possible relationships between morphological and functional properties were explored measuring the kinematics of knee motion and the EMG activation of the RF during unexpected leg dropping. The RF muscle was studied due to its relevant biomechanical role in walking and standing from a functional perspective.

## 2. Materials and Methods

### 2.1. Study Population

Ten adults with DS (five males and five females; mean age, 26.6 ± 4.6 years; age range, 21–34 years) were enrolled in this study. All participants lived in their own home and belonged to the Italian Association for People with Down syndrome (Section of Catania, Catania, Italy). The study participants with DS were regularly involved in physical recreational activities such as dancing. Inclusion criteria were as follows: cytogenetic diagnosis of DS, aged between 18 and 40 years. Exclusion criteria were as follows: severe behavioral disturbances; previous trauma to the thigh; presence of atlantooccipital and atlantoaxial dislocations with spasticity; presence of a cardiac pacemaker; recent fever or convulsions; and severe concomitant diseases. Ten typically developed (TD) adult volunteers (five males and five females; mean age, 23.9 ± 5.0 years; age range, 20–32 years) were recruited to determine the reference values of the examined parameters.

Prior to US examination, a standard physical examination was performed on all participants to determine general health status; this included measurements of weight, height, and calculation of body mass index (BMI). In addition the distance from lateral femoral condyle to the ground, with the subjects in the standing position, was measured to obtain the length of the leg-foot complex.

Local ethics committee of the University of Catania approved the study. In accordance with the Declaration of Helsinki, prior to their inclusion in the study, written informed consent was obtained from the parents or guardians of persons with DS and from the subjects of the control group.

### 2.2. Ultrasonographic Measurements

B-mode US images (Sonosite Titan, Sonosite Inc., Bothell, Washington, USA) using a 7.5 MHz linear probe of the RF muscle of the right thigh were captured when the subjects were completely relaxed on an examination table with legs fully extended. A generous amount of US gel was applied to prevent skin impressions. The ultrasonographic images were obtained at approximately 60–70% of the thigh length from the popliteal crease to the greater trochanter corresponding to the muscle belly of RF. To obtain standard measurements, the probe was placed perpendicular to the muscle surface and adjusted to obtain the brightest image. The US parameters gain and dynamic range were kept at fixed values, whereas only depth was altered to visualize the entire RF. The scan depth was set from 3.9 to 5.5 cm, depending on the size of the muscle mass. Three US scans for each subject were acquired along the longitudinal axis of the RF by an examiner with the consensus of a second examiner. This procedure minimized the discomfort and permitted performing the examination in the subjects with DS in whom the compliance was limited due to mental retardation.

Pennation angle (PA), muscle thickness (TK-M), and thickness of the subcutaneous tissue (TK-S) were measured in the sagittal axis. Pennation angle is the positive angle between the deep fascia and the line of muscular fascicle. Muscle thickness is the distance between the superficial fascia and deep fascia. Subcutaneous tissue thickness is the distance between the skin and the superficial fascia.

Three US scans were acquired and the images with largest and smallest values were excluded from further analysis. All US images were downloaded to a compatible personal computer using the SiteLink Image Manager 2.2 software and IrfanView image viewing software (Sonosite Inc.). Analysis of US images was performed using the Image J analysis software (Research Services Branch, NIMH, Bethesda, MD, USA).

### 2.3. Kinematic and Electromyographic Measurements

The analysis of the knee motion was performed using the pendulum test (for an extensive description of this procedure see Casabona et al., [13]). By means of this technique, angle amplitude variations at the knee were recorded by an electrogoniometer placed on the lateral side of the tested limb. Ten trials were executed with the participants sitting on an examination table, with the trunk inclined approximately 40° from the horizontal to obtain a comfortable starting position. For each trial, the examiner lifted the limb extended with the knee as straight as possible, releasing it so that it could reach to resting position, after passive swings.

Kinematic data obtained by electrogoniometer were low-pass filtered with a zero-lag second-order Butterworth filter with 5 Hz cutoff frequency.

Electromyographic (EMG) data were collected using surface electrodes placed over the rectus femoris: the EMG signal was amplified and sampled at 1 kHz and then, off-line, it was full wave rectified and high-pass filtered (20 Hz).

All the measurements were sampled at 1 kHz and recorded with a portable device (Pocket EMG by Bioengineering Technology and System, BTS, Milan, Italy). Kinematic data were resampled at 200 Hz for further processing.

Kinematic and EMG measurements were elaborated to obtain the following parameters:

(i) Onset angle (OA) corresponding to the angle at rest before the onset of the first flexion.
(ii) Angle of reversal at the end of the first flexion (*F*1).
(iii) Amplitude of the first flexion (*F*1_amp_):  *F*1_amp_ = *F*1 − OA.
(iv) Peak angular velocity of knee movement during the first flexion (peak velocity).
(v) EMG area during the first flexion obtained by computing the integral of the filtered EMG signals (low-pass filter: 2nd-order Butterworth filter with a cutoff of 10 Hz).

### 2.4. Statistical Analysis

Means and standard deviations for the two groups of participants were computed. Preliminary tests for normality (Shapiro-Wilk test) and for equality of sample variances (Levene's test) provided the basis for using a parametric statistics. The independent samples *t*-test was used to compare differences in the mean values of the two groups. The level of significance was set at *P* < 0.05. To validate the statistical outcomes, the effect sizes were estimated by using Cohen's *d*_*s*_ and Hedges' *g*_*s*_, which describe the standardized mean differences between two sets of independent measurements based on sample average. The Hedges' *g*_*s*_ represents a correction of Cohen's *d*_*s*_, since the latter provides a biased estimate of the population effect size. This correction is particularly crucial when small samples are compared. In addition, to improve the readability of the effects size, we computed the Common Language effect size (CL) statistic, which converts the effect size into a percentage and expresses the probability that a randomly selected data from one group will be greater than a randomly selected data from the other group. The effect size computation was based on the recommendation reported by Lakens [15].

To determine the relationships between morphological and functional parameters, we used the following multivariate linear model:

(1) $$\begin{array}{c} \text{FP}=\beta_{0}+\beta_{1}\cdot \text{PA}+\beta_{2}\cdot \text{TK-M}+\beta_{3}\cdot \text{TK-S}+\varepsilon , \end{array}$$

where FP is each of functional parameters (*F*1_amp_, peak velocity, and EMG area), *β*_0–3_ is the standardized partial regression coefficients, PA, TK-M, and TK-S are the three morphological parameters, and *ε* is the residual error.

We computed the coefficient of determination (*R* ^2^), to evaluate the total variance explained by each regression. The specific influence of each independent variable on the depended variable was measured by the standardized partial regression coefficients (*β*_0–3_). In addition, the partial coefficient of determination (*r* ^2^) was computed to determine the portion of variance explained by a given predictor when the other predictors are held fixed. *r* ^2^ was computed as the following:

(2) $$\begin{array}{c} r^{2}=\frac{{\text{RSS}}_{p}-{\text{RSS}}_{t}}{{\text{RSS}}_{p}}, \end{array}$$

where RSS_*p*_ is the residual sum of squares for the model with all but the single predictor of interest included and RSS_*t*_ is the residual sums of squares for the model with all predictors.

Statistical analysis was performed using SYSTAT, version 11 (Systat Inc., Evanston, IL, USA).

## 3. Results

Persons with DS were significantly shorter than the control group (148.3 ± 5.3 cm versus 166.6 ± 9.8 cm, resp.; *t* = 5.21, *P* < 0.0001). Body weight was observed to be moderately lower in the DS group than in the adult TD group (61.4 ± 8.1 kg versus 62.5 ± 11.8 kg, resp.; *t* = 0.23, *P* = 0.8170). The BMI was significantly higher in the DS group than in the control group (28.2 ± 5.2 versus 22.3 ± 2.5, resp.; *t* = 3.21,  *P* = 0.005).

The anthropometric data were correlated with each parameter to justify possible normalization process. *F*1_amp_ was the only parameter which showed a significant correlation with the height (*r* = 0.62; *P* < 0.01) and the length of the leg-foot complex (*r* = 0.69; *P* < 0.01). Considering that *F*1_amp_ is a measure of the knee angle, it was appropriate to normalize this parameter with respect to the length of leg-foot complex.

### 3.1. Morphological Parameters

**Figure 1:**
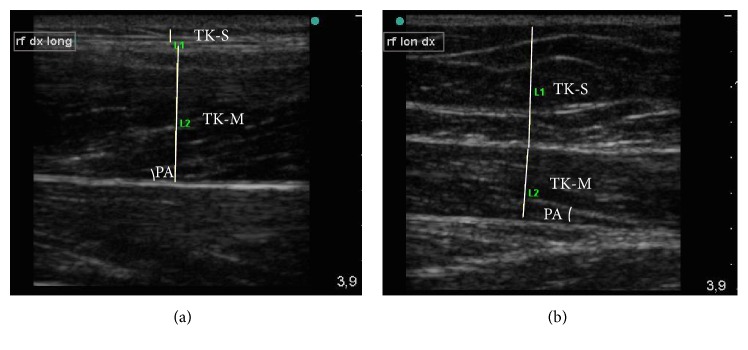
Examples of ultrasound images of the rectus femoris. Comparative evaluation of the muscle rectus femoris (RF) in the sagittal view. (a) Ultrasound image of the RF from a typically developed subject. (b) Ultrasound image from a subject with Down syndrome. TK-S, subcutaneous layer thickness (L1); TK-M, muscle thickness (L2); PA, pennation angle between the fascia and the muscle fascicle.

Figure 1 depicts representative US scans of the RF in a TD adult (Figure 1(a)) and in a person with DS (Figure 1(b)). Greater subcutaneous tissue thickness and reduced muscle thickness can be observed in the person with DS, and the pennation angle was similar in both the DS person and the TD person. From a qualitative perspective, hyperechoic connective tissue septa were found to be dispersed within hypertrophic fatty lobules in the subcutaneous tissue.

**Figure 2:**
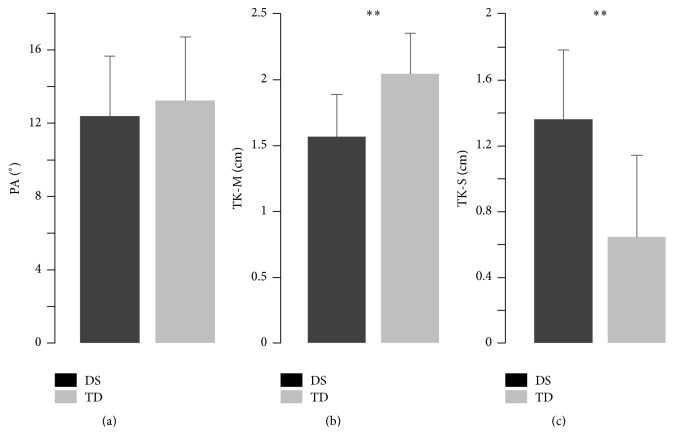
Summary of the descriptive statistics for the morphological parameters. Differences between persons with Down syndrome and typically developed concerning pennation angle (PA), muscle thickness (TK-M), and subcutaneous thickness (TK-S). Data are represented as means and ±SD. ^*∗∗*^ *P* < 0.01.

Table 1 and Figure 2 summarize the descriptive statistic for the morphological parameters. Values of the pennation angle were found to be similar in both the DS group and the TD adult group (Figure 2(a)), whereas muscle thickness was significantly reduced in the DS group (Figure 2(b)). Subcutaneous tissue thickness was found to be greater in persons with DS (Figure 2(c)) due to fat accumulation under the skin and around the thigh, covering the muscle.

### 3.2. Functional Parameters

**Figure 3:**
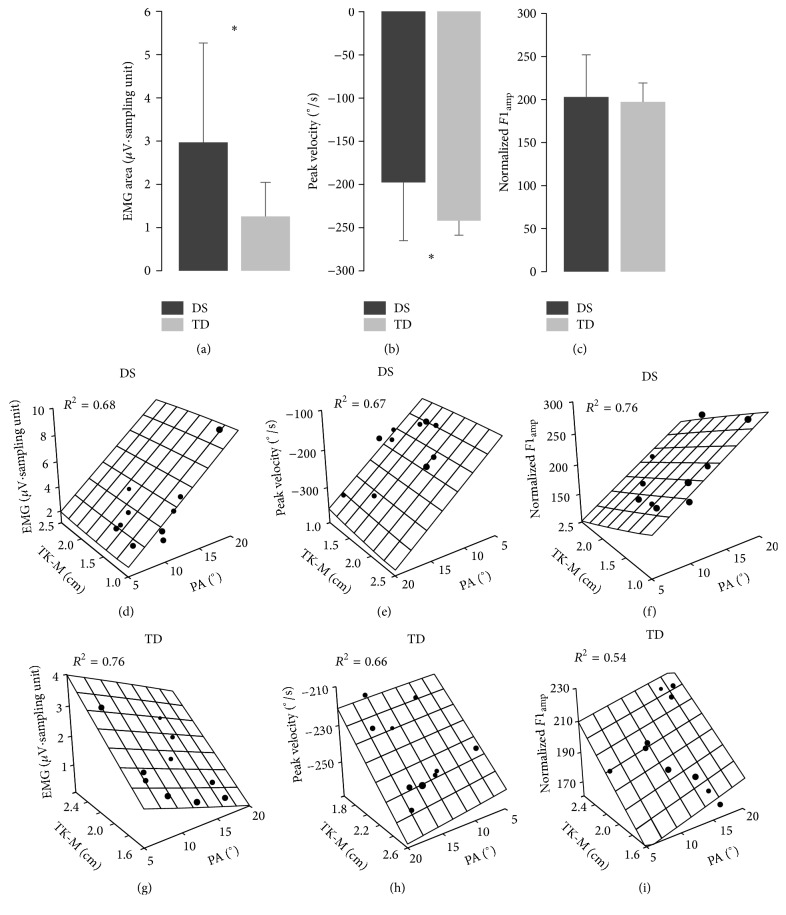
Descriptive statistics for the functional parameters and results of the multivariate regression analysis. Comparisons between persons with Down syndrome and typically developed concerning normalized EMG area (a), peak velocity (b), and *F*1_amp_ (c). Data are represented as means ± SD, ^*∗*^ *P* < 0.05. Planar surface grids, representing the model resulting from the multivariate regression analysis, are superimposed to the observed data (black dots) in persons with DS (d–f) and in TD subjects (g–i). Each regression includes also the subcutaneous thickness (TK-S) as reported in Table 2. However, for clearness of illustration in each plot the morphological parameters (PA and TK-M) that showed a significant relationship with the functional parameter are depicted. *R* ^2^, coefficient of determination for the entire model; other abbreviations as in Figure 2.

Comparing persons with DS with participants TD, the former showed significant higher values of EMG area (*t* = 2.23; *P* = 0.0385; Figure 3(a)) and lower value of peak velocity (*t* = 2.12; *P* = 0.0478; Figure 3(b)). There was no significant difference for the normalized *F*1_amp_ (Figure 3(c)).

The relationships between morphological and functional parameters are illustrated in the Table 2 and Figures 3(d)–3(i). The regression of each functional parameter against the three morphological parameters showed a good level of coefficient of determination (*R* ^2^ ranged from 0.54 to 0.76). However, the partial regression analysis showed that the influence of each morphological parameter on the functional parameters changed in persons with DS with respect to TD persons. In fact, in the persons with DS the PA was the only significant parameter (*P* < 0.05), explaining most of the variance for all the functional parameters (*r* ^2^ ranged from 0.59 to 0.63). Instead, the TK-M was the only significant parameter in determining the changes in the functional parameters in the TD persons (*P* < 0.05; *r* ^2^ ranged from 0.51 to 0.64). This behavior is represented in the plots of Figures 3(d)–3(i), where the linear model (planar surface grids) is superimposed to the observed data for the PA and TK-M. The side of the planar surface with the largest inclination indicates the parameter with the highest standardized beta coefficient and, thus, the parameter with the largest influence on the dependent variable. Over the three functional parameters, the largest slope is exhibited along the PA axis for the persons with DS (Figures 3(d)–3(f)), while, for the TD persons, the axis representing the TK-M showed the largest variations (Figures 3(g)–3(i)).

## 4. Discussion

In this study, the pennation angle, an important architectural spatial parameter associated with the generation of muscle strength, was within physiological limits in persons with DS, whereas muscle thickness was reduced compared with TD adults. Furthermore, thickness of the subcutaneous tissue of the thigh surrounding the RF significantly increased in the group of persons with DS. The functional parameters correlated with pennation angle in persons with DS and with the thickness of RF in TD participants.

### 4.1. Changes in Morphological Parameters

The reliability and validity of US measurements have been demonstrated in several human studies for most lower limb muscles (reviewed by Kwah et al. [16]). Ema and colleagues [17] performed a study in cadaveric and* in vivo* muscle to investigate the validity and applicability of ultrasonography as a method to measure the muscle architecture of the RF. In their study, the measurements were found to be as valid as those for other muscles. In spite of the physiological importance of the RF in postural control and walking, only a few studies looked at pathological changes using ultrasonography. In a study investigating the resting architectural characteristics of the RF muscle in children with diplegia due to cerebral palsy, partially altered muscle architecture with a decrease of the fascicle length and muscle size was observed, whereas there was no change in the pennation angle [18].

In our study, the pennation angle was similar in both the DS and TD adult groups, indicating that spatial organization and orientation of muscle fibers were not abnormal in these persons with DS. From a physiological perspective, the pennate muscle may increase the number of fibers into a given length by orienting the fibers obliquely to the central tendon, with the consequence of having a larger cross-sectional area and a relatively larger capacity for generating high forces in respect to fusiform muscles. Our results suggest that a basic mechanism to increase muscle strength is well represented in the muscles of persons with DS as opposed to TD adults.

We also observed a significant reduction in muscle thickness, signifying reduced muscle volume in persons with DS. In this respect, US measurements of muscle thickness are reliable [19] and may predict the loss of muscle mass in middle-aged and older adults [20]. A reduction in muscle thickness is compatible with muscle weakness observed in DS [2, 3].

DS is a progeroid syndrome characterized by skin atrophy and sclerosis [21]. Alterations of the fetal extracellular matrix were demonstrated in DS in US studies [22], leading to the conclusion that there is an abnormal form of collagen VI and increased hyaluronic acid content that leads to interstitial edema that also occurs in the nuchal region (nuchal edema). A literature search did not reveal any reports on the architecture and composition of subcutaneous tissue in DS. In the current study, we observed a significant increase in the subcutaneous layer (*Hypoderma* and derma) in persons with DS, with a mean thickness value that was almost double the mean value of TD adults. This increase in the layer of subcutaneous fat may be due to overweight or obesity that are frequently found in people with DS. Indeed, most of the study participants with DS were overweight or obese. Obese or overweight persons are generally stronger than normal or underweight individuals; this is most likely due to the fact that obesity causes a loading effect on muscles, namely, antigravity muscles [23]. Recent studies show that obesity or overweight have a different effect on muscle architecture according to age. In young obese girls, excess body mass serves as a chronic training stimulus responsible for an increase in isokinetic muscle strength, showing a positive correlation with an increased pennation angle, muscle thickness, and muscle size [24, 25]. Contrarily, older overweight and obese women were unable to develop a similar adaptive behavior, with the exception of an increased pennation angle [24]. In our study, the majority of participants in the DS group were overweight or obese and a similar adaptation was not found. The pennation angle did not differ from that of TD individuals, and reduced muscle thickness was observed. As previous studies suggest, the changes observed in overweight or obese persons may be considered adaptive changes under the influence of the nervous system [24]. This adaptive mechanism was not observed in participants with DS in our study, possibly due to metabolic or neural maladaptive behavior.

### 4.2. Relationships between Morphological and Functional Parameters

The regression analysis performed to relate the changes in morphological parameters to the functional adaptations during pendulum leg motion provides suggestions on how morphological structure may influence the muscular activation.

The pennation angle showed a good correlation with the variations of joint kinematics and of RF EMG only in the persons with DS. This means that the pennation angle may be considered the morphological correlation of the phasic activation of the RF after the leg release. In previous papers we showed that this muscle activity appears when persons with DS pass from adolescence to the adult age, indicating a possible functional compensation for the inherent ligaments laxity [13, 14]. Thus, changes in pennation angle may be a structural sign associated with specific functional adaptations occurring in DS when rapid muscle activations are required.

On the other hand, the muscle thickness was correlated with the functional parameters only in the control group. Persons TD show a stable tonic muscle activity during the leg pendulum motion [13]; thus, the muscle thickness could influence mainly the maintenance of the muscular tone.

Finally, no significant relationship was found for the subcutaneous thickness in both the groups. In this case, a straightforward interpretation could be that the marginal structural connection between the subcutaneous tissue and muscle fibers prevent possible functional correlations. However, investigating whether the abnormal thickness of the subcutaneous layer may interfere with force transmission from muscle to surroundings tissues may be of interest.

## 5. Conclusions

The ultrasonographic data reported in this study demonstrate, for the first time in persons with DS, that the structural architecture of the RF is within the physiological range (pennation angle), while a reduction of muscle thickness and an increase of subcutaneous layer are observed.

The specific relationships described between morphological and functional parameters provide supports in considering the variability of the pennation angle as a resource used by the persons with DS to adapt the muscular activity when rapid changes occur, such as during walking or other cycling movements. Instead, the reduction in muscle thickness may influence more specifically the muscle tone, producing the typical hypotonia and weakness, which impair the adaptation of postural task, such as maintaining the upright standing.

## Figures and Tables

**Table 1:** Descriptive statistic for the morphological parameters.

	TD	DS	*P*	Cohen's *d*_*s*_	Hedges' *g*_*s*_	CL (%)
PA (°)	13.2 ± 3.5 (7.4–17.0)	12.4 ± 3.3 (8.0–18.4)	0.5834 *t* = 0.56	0.25	0.24	57
TK-M (cm)	2.0 ± 0.3 (1.6–2.5)	1.6 ± 0.3 (1.1–2.1)	0.0033 *t* = 3.38	1.51	1.45	86
TK-S (cm)	0.7 ± 0.5 (0.2–1.5)	1.4 ± 0.4 (0.5–2.0)	0.0027 *t* = 3.47	1.55	1.49	86

Data are represented as means and ±SD with ranges listed in the brackets. TD persons typically developed; DS, persons with Down syndrome; PA, pennation angle; TK-M, muscle thickness; TK-S, subcutaneous thickness; CL, Common Language effect size.

**Table 2:** Relationship between morphological and functional parameters: summary of multivariate regression analysis.

Persons with DS	PA	TK-M	TK-S	*R* ^2^	*r* ^2^
EMG area	0.82^*∗*^	0.03	0.23	0.68	**0**.**63**
Peak velocity	−0.82^*∗*^	−0.09	−0.3	0.67	**0**.**62**
*F*1_amp_	0.66^*∗*^	−0.32	−0.36	0.76	**0.59**

Persons TD					

EMG area	−0.3	0.69^*∗*^	−0.28	0.76	**0.64**
Peak velocity	−0.15	−0.78^*∗*^	−0.56	0.66	**0.62**
*F*1_amp_	0.31	0.73^*∗*^	0.17	0.54	**0.51**

In the rows are reported the standardized regression coefficients for each regression. In bold are represented the significant independent variables (^*∗*^ *P* < 0.05) and their fractioned contribution to the variance of the dependent variable (*r* ^2^). *R* ^2^ is the coefficient of determination for the entire model.
